# Influence of international industrial transfer on the structural power of global value chain -Empirical evidence from manufacturing in RCEP Countries

**DOI:** 10.1371/journal.pone.0291973

**Published:** 2024-03-07

**Authors:** Yu Zhang, Lee Joohyeong, Minjian Qiao, Minglong Kou

**Affiliations:** 1 School of International Politics and Economics, University of Chinese Academy of Social Sciences, Beijing, China; 2 School of Economics, Hebei University, Baoding, China; 3 School of Economics and Management, Xinjiang University, Urumqi, China; Nanjing University of Information Science and Technology, CHINA

## Abstract

International industrial transfer (IIT) has spawned changes in the deep-seated structural power of value-added. We creatively construct an IIT index which includes both scale and direction, and constructs the structural power (SP) of value-added based on added value. Furthermore, based on 15 RECP countries from 1995 to 2018, this study uses a two-fixed-effect regression model to investigate the IIT on the structural power of value-added of RCEP countries. The results show that: (1) IIT can significantly promote the structural power of value-added of RCEP countries, and the benchmark regression conclusion is still valid after a series of robustness tests; (2) Heterogeneity analysis shows that IIT has a more significant promoting effect on the structural power of value-added in developed countries than in developing countries and a more significant promoting effect on the structural power of value-added inward than on the structural power of value-added outward; (3) Intermediary mechanism test shows that IIT mainly affects the structural power of value-added through trade openness and foreign direct investment. The conclusions of this paper provide useful enlightenment for enhancing the structural power of manufacturing value-added in RCEP countries in the context of global value chain division.

## 1. Introduction

Academics Four large-scale international industrial transfers (IIT) have profoundly changed the global economic pattern. Among them, the third and fourth international industrial transfers focus on East Asia and Southeast Asia, which promote the economic rise of eastern Asia. Firstly, “The Four Asian Tigers” emerged, then followed by “Tiger Cub Economies” and China. The eastern region of Asia has gradually become one of the world’s most active economic regions. Compared with the previous three international industrial transfers, the most obvious feature of the fourth international industrial transfer is the transfer from the complete product value chain to the processing value chain [1]. Since the 1980s, global value chain trade has gradually increased in international trade, and the deep network structure of globalization shaped by the transnational flows of global value chains has generated structural power. The position and influence of a country in the global value chain network determine its size of structural power, which is very important for the understanding of international relations in the context of the global value chain.

The Regional Comprehensive Economic Partnership Agreement (RCEP) officially entered into force on January 1, 2022 [2]. After ten years of implementation, the members have successively ratified the implementation agreement, marking the formal implementation of the largest and most potential free trade area in the world. The entry into force of RCEP means that, in the current international environment where anti-globalization and trade protectionism prevail in European and American countries, regional integration in eastern Asia has been strengthened. Additionally, even the degree of trade openness and facilitation in eastern Asia has been further improved, which is of great significance for stabilizing regional industrial supply chains, promoting regional value chain integration, better coping with the risk of chain break in developed countries, and promoting regional and world economic development [3]. However, the internal economic development levels of RCEP member countries are uneven, including highly developed countries, developing countries and underdeveloped countries. Due to the differences in economic development level, geographical location, and resource endowment, the economic specialization mode and competitive advantage of each member country are also different. Ito et al. (2017) [4] and Fernandes et al. (2020) [5] empirically found that traditional factors such as factor endowments and geographical location not only significantly affect the patterns a country participates in the global value chain but also determine its position and structure power of the global value chain. RCEP is the world’s largest and most potential free trade zone and an important undertaker of international industries. The unevenness of its economic development and the difference in the mode of economic specialization provides a natural experimental group for studying the country’s changes in the structural power of added value caused by IIT. The RCEP sample selection is importantly typical and representative. RCEP has always been one of the most important places to undertake international industrial transfer, and it is also one of the most economically active and important regions in the world. Firstly, according to the data of the World Bank, the total amount of FDI increased significantly from US$ 81.40 billion in 1995 to US$ 540.70 billion in 2022, with an average annual growth rate of 7.0%. RCEP’s share of world FDI has grown rapidly from 22.5% in 1955 to 29.5% in 2022. Secondly, the GDP of the RCEP region increased significantly from US$ 7.95 trillion in 1995 to USD 29.40 trillion in 2022, with an average annual growth rate of 4.8%. The proportion of GDP rose rapidly from 25.6% in 1995 to 29.1% in 2022. Therefore, the study area and sample are highly representative. Based on this, this paper attempts to use the TIVA database (OECD-TIVA 2021) to measure the structural power of RCEP countries and the scale of IIT and analyze the impact and mechanism of IIT on the structural power of RCEP countries in the value-added network.

## 2. Literature review

### 2.1 Structural power

In the 1980s, Caporaso(1978) [6], an expert in international political economy and international relations theory at the University of Washington, regarded structural power as “the ability to manipulate the choices, strengths, alliance opportunities and benefits that actors may use”. It is different from “decision-making power” and “bargaining power” that controls the outcome of specific events. It is a higher-order form of power that can control bargaining power and rules. However, the traditional analysis of the concept of power ignores the important power phenomena at the level of strategic interaction and bargaining. Many researchers have begun to criticize the concept of power in neorealism and try to expand the scope of power analysis, including exploring structural power [7]. The most representative research is the theoretical framework of structural power established by Strange (1994) [8], which systematically analyzes the interaction between the market and the state. During the same period, Gill and Law (1989) [9] focused on the structural power of global hegemony and capital, believing that such hegemony is unsustainable. Guzzini (1993) [7] summarized the three characteristics of the concept of structural power: indirect institutional, unintentional, and non-personal creativity, and pointed out that any power analysis should include one or two pairs of power concepts, linking agent power and non-personal governance. In Guzzini’s view, the structural power interpreted by Strange has the attributes of “indirect system” and “unintentional”; the structural power understood by Caporaso (1978) [6], Gill and Law (1989) [9] has the attributes of “indirect system” and “impersonal creation”. At this point, the research path of structural power theory has begun to take shape.

Structural power mainly refers to the social ability and interests of the actors in the structure, in which the structure is regarded as an internal and direct relationship. With the integration of disciplines, the development of social network analysis [10], which has deep theoretical roots, has greatly enriched the connotation of structural power and expanded the micro-foundations for understanding it [11, 12]. The essence of the theoretical method of social network analysis is the method of “structural analysis”, which combines the two research paths of structuralism and constructivism, and provides a new way of thinking for understanding structural power. It provides a unique microscopic perspective on the meaning of dynamic and complex network structures for individual actions and social structures, as well as the interaction between actors and between actors and structures. Inspired by this method, structural power includes not only the indirect influence exerted by actors on specific actors through “structure”, but also the direct interaction between actors.

### 2.2 Structural power of global value chains

The concept of global value chain evolved from the early concept of “commodity chain” [13]. The commodity chain focuses on tracking the whole process of how the components of goods are transformed into final consumer goods step by step. Since the 21st century, the “value chain” has replaced the “commodity chain”, unifying the two processes of trade and production into the creation, flow, and distribution process of “value added”. The global value chain is the simultaneous fragmentation of production activities in both functional and geographical aspects, which is formed, expanded, and deepened through the exchange and flow of tangible products and intangible knowledge and technology [14]. These chains are not parallel, but closely intertwined, forming a complex network throughout the world, in which power can be generated and operated. It is the core task of understanding and judging the international pattern to explore the form and essence, distribution and growth and decline of power in the international system. When we look at the international pattern in the dimension of economic globalization, we cannot avoid analyzing the nature and characteristics of power in the global value chain.

Value added refers to the value created by labor and equipment processing, intellectual input, and circulation marketing, which is more than the value of raw and auxiliary materials, and can be created and distributed in the production and circulation links. In other words, the value added in the global value chain is the “gain” in transnational collaboration and exchange relations. Value added is the ultimate meaning and unique feature of global value chains, which is related to the gains and losses of participating in globalization, and its creation, flow and distribution are also closely related to power. In the discussion of relational power, international relations scholars have long called for the study of interdependence in the gain dimension, and take trade as an example to emphasize that the “micro-theory” that establishes a logical relationship between exchange and power does not depend on the face value of trade, but on the trade gain [15]. As an asymmetric sensitivity and vulnerability of the source of relational power, it is essentially an asymmetric gain fluctuation range and degree of damage. Although the value-added flow is based on the import and export exchange relationship of intermediate goods, it does not start and end with bilateral trade, but continues to flow and increase in the connectivity network until it is finally consumed [16]. Therefore, the value-added relationship is not limited to the first-order direct exchange, but includes the high-order exchange relationship throughout the whole network, that is, the value-added network connection. Even if there is no bilateral trade, countries may also have a high degree of value-added interdependence through network connectivity, resulting in mutual influence with structure as the medium. Therefore, structural power arises from the networked dependence on the gain dimension formed by the flow of value added in the global value chain, which is different from the “relational power” in the binary interdependence.

### 2.3 Research on the measurement of international industrial transfer (IIT)

Industry transfer mainly has two dimensions: time and space. Lots of research on its measurement methods have been carried out, which can be divided into relative and absolute measurement methods.

First, is the research on the relative measurement of industrial transfer. Maria Savona et al. (2004) [17] calculated the impact of IIT on Italian industrial linkages based on the principle of location entropy. Li et al. (2018) [18] comprehensively use the Herfindahl index to measure the IIT pattern and direction of labor and capital-intensive industries in China. Xie et al. (2018) [19] analyzed the spatial transfer of various industries in the Yangtze River Delta region according to the change of the highest market share in the province of China. Liu (2015) [20] constructed an industrial gradient coefficient based on the location entropy and studied the key industries of industrial transfer. The above scholars added other measurement methods based on location entropy to depict industrial transfer but failed to consider that the expansion of market share in a region may come from its economic growth. Sun et al. (2018) [21] considered this influencing factor, constructed an industrial transfer measurement method to eliminate natural growth, and characterized the spatial characteristics of labor, capital, and technology-intensive industrial transfer among Chinese provinces.

Second, is the research on the absolute measurement of industrial transfer. Scholars mostly regard FDI or OFDI as a proxy variable of the input and output of IIT and study the impact from both positive and negative aspects. Zhang and Chen (2020) [22] empirically analyzed the impact of foreign industrial transfers on China’s trade structure, they found that pro-gradient OFDI triggered the transfer of primary industries and reduced China’s exports, and the counter-gradient OFDI will increase the level of China’s manufacturing output. Li et al. (2019) [23] found that FDI provides an opportunity for the development of China’s modern service industry and promotes positive effects such as adjusting the industrial structure in the eastern region through “vacating the cage for birds”, and also had negative effects such as technological repression and low-end locking of global value chain. Jiang et al. (2020) [24] analyzed the impact of OFDI on the upgrading of industrial structure. The empirical results found that OFDI significantly promoted the upgrading of industrial structure and that there were significant lag effects and spatial spillover effects. Hao et al. (2020) [25] incorporated FDI and OFDI into the same framework to study the relationship between the interaction mechanism and the quality of economic growth. The study found that the interaction promoted technological progress and labor factor allocation efficiency, thus promoting high-quality economic growth. Tang et al. (2020) [26] found that IIT is beneficial to promoting the development of secondary and tertiary industries, which optimized China’s industrial structure. Razzaq et al. (2021) [27] found that China’s OFDI to countries along “the Belt and Road” will enhance green economic growth, mainly through improving labor mismatch, upgrading industrial structure, and increasing R&D investment. Li et al. (2022) [28] found that domestic industrial transfer promotes the eastern region’s industrial structure upgrading, but was not conducive to the western regions. Wang and Li (2020) [29] found that FDI has dual characteristics of “pollution shelter” and “pollution halo”. Yan and Peng (2014) [30] take 12 provinces and cities in the eastern region as research samples to study the effect of the OFDI industry hollowing out. The results show that OFDI is the main influencing factor in the OFDI industry hollowing out in Guangdong, Shanghai, and other regions with large foreign direct investment stock. Song et al. (2020) [31] studied the impact of FDI on the environment in 253 cities in China and found that FDI worsened the environmental governance in the middle-income stage and brought great environmental pressure to China. Chen et al. (2017) [32] calculated the impact of FDI on China’s wage gap based on China’s industrial enterprise database. The study found that FDI and the host country’s enterprise wage gap showed an inverted U-shaped relationship.

However, FDI or OFDI can only represent the input and output of IIT in a narrow sense. Just as scholars Zhang and Liu (2009) [33] expanded the existing inter-product industrial transfer to intra-product industrial transfer, focusing on the changes in the spatial distribution of specific processes. The form of industrial transfer has changed, and the change in production location is difficult to reflect IIT. Therefore, the applicability of the above methods is generally limited. Liu et al. (2011) [34] used the input-output model for the first time to quantitatively measure the scale and characteristics of China from 1997 to 2007 and measured and analyzed the domestic industrial transfer from the generalized industrial transfer level. Wang and Wu (2017) [35] further used the input-output model to measure the scale of IIT. The study found that OECD countries tend to transfer some labor and resource-intensive manufacturing industries to non-OECD countries, while the trend in the service industry was not obvious. Zhao and Yin (2011) [36] estimated the industrial transfer by comparing the relative changes in total manufacturing output value between the base period and the current period. The above method makes up for the shortcomings of international industrial transfer in a narrow sense, and can effectively measure the status of a country’s industrial sub-international market, which is also widely used in international industrial measurement.

In summary, the existing research focuses on Structural Power, Structural Power of Global Value Chains, International Industrial Transfer, but there are still the following lacks: (1) The existing literature on the structural power of global value chains mainly focuses on the national level, while there are relatively few discussions on regional scales such as free trade zones. (2) Existing structural power and structural power of global value chains are mostly conceptualized and scoped, with few quantitative studies followed by comparative analysis. (3) Previous studies on the structural power of global value chains mostly focus on “power transfer” to ignore its influencing factors, most previous studies of structural power in global value chains have focused on “power shifts” and neglected their influencing factors, especially IIT as an important influencing factor affecting international relations.

Compared with previous studies, the marginal contribution of this paper is reflected in the research on the relationship between IIT and the structural power of the global value chain. This paper details three aspects; firstly, we creatively construct an IIT index that includes both scale and direction, and constructs the index of the structural power of value-added, which enriches the research on IIT and global value chain structural power. Secondly, from the perspective of research, IIT is an important factor affecting the status change of the global value chain that produces the structural power of added value. This paper incorporates the IIT and the structural power of added value into a unified analytical framework to analyses the impact of IIT on the structural power of added value. Lastly, in terms of mechanism test, this paper further tests this mechanism after clarifying the impact of IIT on the structural power of value-added, providing path support for how to consolidate and deepen the impact of the industrial transfer on the structural power of value-added.

## 3. Research design

### 3.1 Quantitative measurement of the international industrial transfer index

The Industrial transfer is a dynamic and gradual development process, which usually includes two types of transfer within a country and between countries. When the industrial transfer occurs transnational behavior, is called international industrial transfer. There are many factors of international industrial transfer, such as changes in the economic structure of a country, environmental protection, changes in labor capital elements, and so on. Industrial transfer in this paper refers to the change of industrial production share in location after deducting its demand growth in a certain period. Zhao and Yin (2011) [36] estimated the industrial transfer by comparing the relative changes in total manufacturing output value between the base period and the current period. The specific calculation formula is as follows:

$$TR_{\text{ci},t}=\frac{q_{ci,t}}{\sum_{c=1}^{n}q_{ci,t}}-\frac{q_{ci,t_{0}}}{\sum_{c=1}^{n}q_{ci,t_{0}}}$$ (1)

Where *TR*_*ci*,*t*_ represents the transfer degree of industry *i* in region *c* in year *t*, *q*_*ci*,*t*_ represents the total output value of industry *i* in region *c* of year *t*, *n* is the number of regions, $\sum_{c=1}^{n}q_{ci,t}$ representing the overall output value of industry *i*.

### 3.2 The Structural power of value-added (SPV)

The traditional trade accounting method is based on the final exporting country or region, but this statistical method is difficult to apply in the context of globalized production. The division of labor in the global value chain has become the norm of the current global mass production. Each country undertakes the production of some production links. According to the traditional presidential accounting of trade, there are a lot of duplicate statistics, which will seriously exaggerate the value of products in a country’s exports. Johnson & Noguera (2012) [37] and Koopman (2012) [38] found that the Sino-US trade deficit was reduced by 1/3 from the perspective of added value rather than total exports, which means that the traditional total trade accounting exaggerated the value of Chinese exports. Domestic value-added is contained in the export products to measure the economic benefits of a country’s participation in the global value chain, which can objectively reflect the bilateral and even multilateral trade benefits [39]. Therefore, this paper constructs a structural power of value-added to measure this relationship from the value-added perspective.

The structural power of value-added represents the ratio of the output and input-added value of a country to the output and input-added value of all countries in the RCEP network to measure the importance of a country in the RCEP trade network. According to the flow direction of value-added, SPV can be decomposed into two parts, structural power of value-added outward (SPO) and structural power of value-added inward (SPI) [40]. The specific calculation formula is as follows:

SPOjt=∑i≠jTiVXjtji∑j∑i≠jEiXjtji(j∈RCEP,i∈WORLD) (2)

SPIjt=∑i≠jTiVMjtjiΣj∑i≠jEiMjtji(j∈RCEP,i∈WORLD) (3)

Where *SPO*_*jt*_ represents the value-added outward of country *j* to the total exports of all member countries in the RCEP network, ∑i≠jTiVXjtji represents the aggregate domestic value-added of country *j*’s exports, Σj∑i≠jEiXjtji represents the total exports of all member countries in the RCEP network; *SPI*_*jt*_ represent that *j*-country value-added inward account for the proportion of total imports of all member countries in the RCEP network. ∑i≠jTiVMjtji indicates that *j*-country imports contain total foreign value-added, and Σj∑i≠jEiMjtji represents total RECP imports. When the scale of country *j*’s output or input of added value to RCEP countries is larger, its value-added output or input accounts for the larger share of RCEP’s overall exports and imports, which means the greater the structural power of output or input of value-added.

In addition, if country *j* is both an exporter and an importer of value-added in the RCEP network, its overall structural power is the average of the sum of the two structural powers:

$$SPV_{j\text{t}}=(SPO_{jt}+SPI_{jt})/2$$ (4)

### 3.3 Model construction

To examine the impact of IIT on the value-added structural power of RCEP countries, this paper constructs the following model:

$$\begin{array}{c} SPV_{it}=\alpha_{0}+\alpha_{1}T\text{ra}ns_{it}+\alpha_{2}HU_{it}+\alpha_{3}Capital_{it}+\alpha_{4}Labor_{it}+\alpha_{5}RD_{it}\\ \;+\alpha_{6}INS_{it}+\theta_{i}+\mu_{t}+\varepsilon_{it} \end{array}$$ (5)

where subscripts *i* and *t* represent country and year respectively. The explained variable *SPV*_*it*_ represents the structural power index of the global value chain for the *i* country in *t* year; *Trans*_*it*_ is the core explanatory variable, represents the IIT scale of a country in year *t*; important factors affecting the structural power index such as the human capital (*HU*), capital intensity (*Capital*), labor scale (*Labor*), R&D expenditure (*RD*) and institutional quality (*INS*). *θ*_*i*_ a fixed effect of country, *μ*_*t*_ is a fixed effect of time. To examine the impact of IIT on the value-added structural power of RCEP countries, need to pay attention to the coefficient sign and significance of *α*_*1*_ in formula (5).

### 3.4 Variable definition

#### 3.4.1 Explained variable

The explained variable of this paper is the structural power index of added value (*SPV*), which is used to measure the importance of a country in the RCEP global value chain network. It is composed of structural power of value-added outward (*SPO*) and the structural power of value-added inward (SPI).

#### 3.4.2 Explanatory variable

The explanatory variable of this paper is the IIT (*Trans*). The IIT has gone through the development process of inter-industry and intra-industry transfer to intra-product transfer. Especially under the background of global value chain trade occupying the dominant position of global trade, the object form of IIT has changed. The traditional use of FDI as the proxy variable of IIT has great limitations in applicability. Therefore, this paper uses the idea of Zhao and Yin (2011) [36] to measure the scale of international industrial transfer of RCEP countries.

#### 3.4.3 Control variables

(1) Human capital (*HU*). This paper uses the enrollment rate of colleges and universities to measure the level of human capital in a country. Human capital has greater potential and value-added space than physical capital, which is an important way to improve labor productivity and promote economic growth [41].
(2) Capital intensity (*Capital*). It is expressed by the proportion of fixed asset investment in the GDP of each country. Capital is one of the important elements to expand reproduction, and investment in fixed assets is an important way for enterprises to improve production efficiency [42].
(3) The size of the labor force (*Labor*). Labor is expressed by the number of people aged 15–64. As another important type of factor input, labor has an important impact on a country’s industrial structure, export trade, and enterprise innovation [43–45].
(4) R&D expenditure (*RD*). It is expressed by the ratio of R&D investment to GDP. Science and technology are the primary productivity and the source of economic growth, and R&D investment is helpful to promote technological progress [46].
(5) Institutional quality (*INS*). INS is an important source of a country’s comparative advantage and has a heterogeneous impact on the export of products with different factor intensities [47]. There are many methods to measure institutional quality, such as the World Bank’s global governance index, the market index, and the risk guidelines of the world. This paper uses the Global Governance Index to measure the quality of a country’s institutions, covering six indicators—including voice and responsibility, political stability, government efficiency, rule quality, legal level, and corruption control, which can be effectively used for conducting international comparisons.

### 3.5 Data source

The calculation of IIT needs to use the global input-output table. At present, the most widely used data are; the Asian Development Bank Database (ADB), the World Input-output Database (WIOD), the Global Supply Chain Database (EORA), and the Organization for Economic Cooperation and Development Database (OECD -TIVA). The database of the OECD -TIVA has a longer time range, faster update, and is more widely used and thereby this paper uses the latest OECD-TIVA (2021) database to measure the scale of IIT and the structural power index of added-value of 15 RECP countries from 1995 to 2018. Other variable data comes from the World Bank WDI and OECD databases. In this paper, the interpolation method is used to fill in the missing values. The descriptive statistics and definitions are shown in Table 1.

**Table 1:** Statistical description of variables.

Variables	Obs	Mean	Max	Min	S.D
SPV	360	5.7422	38.0734	0.0340	8.5004
Trans	360	0.3176	29.2980	-13.6168	4.8077
HU	360	39.9306	120.9657	1.2649	30.5873
Capital	360	19.1671	44.5246	7.8686	45.1308
Labor	360	66.4037	78.7460	50.4917	5.5192
RD	360	0.9674	4.5275	0.0016	1.0868
INS	360	0.1699	1.8619	-1.7516	0.9966

## 4. Empirical results

### 4.1 Baseline results

Table 2 reports the empirical results of IIT on the structural power of added value. Column 1 considers only the effect of the core independent variable (Trans) on the dependent variable (SPV). The coefficient of Trans means that the increase of Trans significantly promotes a country’s structural power of added value. Columns (2)-(6) gradually add other control variables, and the coefficient of Trans remains positive and statistically significant. The calculation results support the conclusion of this paper that IIT is conducive to enhancing the structural strength of a countries value-added.

**Table 2:** Benchmark results.

VARIABLES	(1)	(2)	(3)	(4)	(5)	(6)
	SPV	SPV	SPV	SPV	SPV	SPV
Trans	0.963*** (35.01)	0.963*** (35.22)	0.955*** (35.20)	1.734*** (47.67)	1.733*** (48.12)	1.747*** (29.02)
Capital	-	0.005** (2.19)	0.060*** (2.76)	0.053*** (4.87)	0.053*** (4.96)	0.053*** (4.90)
RD	-	-	0.517*** (3.25)	0.234** (2.48)	0.452*** (3.13)	0.464*** (3.07)
INS	-	-	-	-0.675* (-1.94)	-0.716** (-2.08)	-0.721** (-2.08)
HU	-	-	-	-	-0.972** (-1.97)	-0.940* (-1.86)
Labor	-	-	-	-	-	-0.015 (-0.29)
Constant	5.4410*** (11.69)	5.3354*** (11.47)	5.2622*** (11.21)	4.1545*** (9.35)	8.1443*** (3.94)	8.971** (2.54)
Country fixed effect	Yes	Yes	Yes	Yes	Yes	Yes
Year fixed effect	Yes	Yes	Yes	Yes	Yes	Yes
Observations	360	360	360	360	360	360
R-squared	0.7927	0.7957	0.8024	0.9614	0.9624	0.9625

Note:

*, **, and *** indicate significance at the 10%, 5%, and 1% levels, respectively. The t values are in parentheses. the same below.

From the perspective of the coefficient symbol and significance level of control variables, firstly, fixed asset investment is an important way for a country to expand reproduction, which is a necessary condition for a country’s economic development. Secondly, the impact of R&D expenditure on the structural power of value added is consistent with the research results of most scholars. R&D investment helps to improve the level of scientific and technological innovation and enhance the international competitiveness of products. The impact of INS on the structural power of value added is significantly negative. As Qiu et al. (2014) [48] found in their study, when institutional quality exceeds a certain threshold, it can promote exports. The possible reason is that the institutional quality level of RCEP countries is within the threshold value, which hinders the improvement of the structural power of value added in the short term. The effect of Labor on the structural power of value-added does not pass the significance test, reflecting that labor force size fails to enhance the structural power of value added in RCEP countries. The impact of Capital on the structural power of added value is not obvious. Human capital is an important way for a country to effectively improve labor productivity, which means that RCEP countries should increase investment in education and improve the level of human capital.

### 4.2 Heterogeneity analysis

#### 4.2.1 The heterogeneity of national development level

Within the RCEP region, there are not only developed countries such as Japan, South Korea, and Australia, but also many developing countries, represented by China. RCEP countries have great differences in factor endowments, economic development levels, and infrastructures. So, these factors have differential impact on how does IIT effect the structural power of value-added respectively. From columns (1)–(2) of Table 3, in both developed and developing countries, IIT significantly effects on the structural power of added value positively, but the impact of IIT on developed countries is greater. The main reason is that the level of economic development, institutional quality, and business environment in developed countries are much higher than those in developing countries, and high value-added industries in IIT prefer developed countries. Developing countries undertake low value-added industries in IIT, which contain a low technical level and value-added content, and create low value-added.

**Table 3:** Heterogeneity test.

VARIABLES	(1)	(2)	(3)	(4)
	Developed	Developing	SPO	SPI
Trans	1.479*** (16.89)	1.407*** (2.73)	2.189*** (33.62)	1.305*** (18.64)
Constant	-30.608*** (-3.21)	17.930** (2.17)	-0.837*** (-0.22)	-18.778*** (4.57)
Control variables	Yes	Yes	Yes	Yes
Country fixed effect	Yes	Yes	Yes	Yes
Year fixed effect	Yes	Yes	Yes	Yes
Observations	120	240	360	360
R-squared	0.9765	0.9854	0.9734	0.9093

#### 4.2.2 The output and input heterogeneity of structural power of value-added

From a global perspective, the RCEP region is a typical export-oriented economy, and the total export trade is much larger than the total import trade. Focusing on the structural power of added value in this paper, IIT will also have a differential impact on the structural power of value-added inward and outward of member countries in the region. IIT has a significant positive role in promoting both the structural power of value-added inward and outward. Among them, the impact of IIT on the structural power of value-added inward is smaller than the structural power of value-added outward.

### 4.3 Robustness test

This paper uses four methods to test the robustness; substitution variable, change estimation methods, eliminating special years, and endogenous test.

#### 4.3.1 Substitution variable

Differences in measurement methods will affect the measurement results of the explained variable. The previous measurement method of the structural power of value-added adopts the arithmetic average. This paper also uses the geometric average method to recalculate the structural power of value-added and replace the original dependent variable. The estimated coefficients and statistical significance tests of the core explanatory variables and other control variables maintain a high logical consistency with the benchmark regression results in Table (3), which verifies that the benchmark regression results are reliable and robust.

#### 4.3.2 Measurement method

To effectively correct the model estimation bias caused by the heteroscedasticity between groups within RCEP countries and the internal autocorrelation of control variables, this paper uses maximum likelihood estimation (MLE) and generalized least squares (FGLS) to re-estimate the model which are shown in columns (2)-(3) of Table 4. The empirical results show that the core explanatory variable IIT is still significantly positive to the dependent variable (SP), and all of them pass the econometric robustness test at the 1% level. In a word, the robustness test based on different measurement methods reconfirms the robustness and reliability of the benchmark model.

**Table 4:** Robustness checks.

Variables	(1)	(2)	(3)	(4)	(5)	(6)
	SPV1	MLE	FGLS	SPV	SYS-GMM	DIFF-GMM
Trans	1.670*** (28.58)	1.747*** (32.75)	1.788*** (27.65)	1.715*** (28.24)	1.899*** (32.14)	1.429*** (4.68)
Constant	4.514 (1.32)	8.971*** (2.87)	14.457*** (6.66)	8.391** (2.34)	4.693 (1.37)	-13.760** (-2.17)
Control variables	Yes	Yes	Yes	Yes	Yes	Yes
Country fixed effect	Yes	Yes	Yes	Yes	Yes	-
Year fixed effect	Yes	Yes	Yes	Yes	Yes	-
Observations	360	360	360	330	360	360
R-squared	0.9640	-	-	0.9641	-	-
LR/Wald	-	629.71	20120.07	-	-	-
AR(1)	-	-	-	-	0.009	0.066
AR(2)	-	-	-	-	0.208	0.333
Sargan/ Hansen Test	-	-	-	-	177.25	7.09

#### 4.3.3 Financial crisis

Considering the impact of the financial crisis on the model estimation and drawing on Cui (2021) [49] approach, this paper excludes two years of 1999 and 2009. The former is resulted from the anomalies of the 1998 Southeast Asian financial crisis and the 2008 global financial crisis. From the test results which are shown in column (4) of Table 4, the IIT coefficient is significantly positive. In brief, the robustness test based on eliminating outliers further shows that the obtained results are robust and reliable.

#### 4.3.4 Endogenous test

Considering that the previous structural power of added value will have an impact on the current structural power of added value which will result some errors in model estimation, this paper uses the system GMM and the difference GMM to re-estimate the model to solve the possible endogeneity problem in the model setting. The regression results and the measurement dominance test are very robust, which are shown in columns (5)–(6) of Table 4. In short, the results again confirm that IIT can indeed improve the value-added structural power of RCEP countries.

## 5. Mechanism analysis

The important carriers of IIT are international trade and international capital flow (FDI). International trade and international capital flows can influence the industrial structure, economic growth, import and export trade, technological innovation, and other influences. These, thereby, further improve the production efficiency and international competitiveness of products in importing countries and ultimately reshape the structural power of value-added. Therefore, this paper further analyzes the potential influence mechanism of IIT on the structural power of added value from the perspective of trade openness and foreign direct investment.

Drawing on the practices of Baron and Kenny (1986) [50], this paper constructs the following model and uses the three-step method to test the mediating effect of whether the international industrial transfer can enhance a country’s structural power of added value through two channels: trade openness and foreign direct investment.


$$SPV_{it}=\beta_{1}+\beta_{2}T\text{ra}ns_{it}+\beta_{2}X_{it}+\theta_{i}+\mu_{t}+\varepsilon_{it}$$ (6)

$$Med_{it}=\gamma_{1}+\gamma_{2}T\text{ra}ns_{it}+\gamma_{2}X_{it}+\theta_{i}+\mu_{t}+\sigma_{it}$$ (7)

$$SPV_{it}=\eta_{1}+\eta_{2}T\text{ra}ns_{it}+\eta_{3}M{\text{ed}}_{it}+\eta_{4}X_{it}+\theta_{i}+\mu_{t}+\omega_{it}$$ (8)

Formula (6) is consistent with the previous benchmark regression model. The coefficient *β*_*2*_ of Trans reflects the aggregate effect of IIT on the structural power of value added at the country level. There are two explained variables in Eq (7), namely, trade openness (*Openness*) and foreign direct investment (*FDI*). If we can observe that the Med coefficient γ_2_ in Eq (7) is significantly positive, that is, IIT can increase a country’s trade openness and foreign direct investment. The coefficient η_2_ of Trans indicates the direct effect of IIT on the structural power of added value, while the coefficient η_3_ of Med indicates the indirect effect of the mediating variable on the structural power of added value after controlling for Trans.

### 5.1 Trade openness

Trade openness generally refers to the degree of openness of a country‘s trade in goods [51]. Its measurement methods are mainly regular and resultative. Trade dependence is the most used measurement for the index of trade openness as the data is easy to obtain and the calculation is simple making it easy for international comparison. It is generally measured by the ratio of a country’s total import and export trade to GDP. This paper also uses this method to measure the trade openness of RCEP countries.

Neoclassical economic growth theory believes that trade openness can effectively promote capital formation, improve resource allocation efficiency, and technological progress, and promote economic growth [52–54]. To verify the impact of IIT on the structural power of value-added by improving the trade openness of RCEP countries, this paper conducts regression on IIT and trade openness, and the results are shown in Column (1) of Table 5. IIT has a significant positive role in promoting trade openness, indicating that IIT can improve the trade openness of importing countries, which further enhances a country’s structural power of added value. The coefficients of β_2_, γ_2_, and η_3_ in the mediating effect model of trade openness in Table 5 (1)–(3) are all significant, so the mediating effect exists. The magnitude of this effect is 0.519 (obtained by multiplying the coefficients γ_2_ and η_3_), which accounts for about 29.7% of the total effect of IIT on the structural power of added value (obtained by dividing the total effect by the mediating effect: γ_2_*η_3_/β_2_). This result suggests that there is a mechanism that exists for IIT to promote the rise of a country’s value-added structural power by increasing trade openness, and this mechanism can explain 29.7% of the total impact of IIT on value-added structural power.

**Table 5:** Mechanism analysis.

Variables	(1)	(2)	(3)	(4)	(5)	(6)
	SPV	Openness	SPV	SPV	FDI	SPV
Trans	1.747*** (29.02)	0.220*** (5.70)	1.483*** (12.95)	1.747*** (29.02)	0.506*** (3.19)	1.613*** (16.68)
Openness			2.358** (2.15)			
FDI						0.242* (3.02)
Constant	8.971** (2.54)	1.014** (2.31)	-54.938 (-1.08)	8.971** (2.54)	14.189*** (8.43)	20.504 (0.52)
Control variables	Yes	Yes	Yes	Yes	Yes	Yes
Country fixed effect	Yes	Yes	Yes	Yes	Yes	Yes
Year fixed effect	Yes	Yes	Yes	Yes	Yes	Yes
Obs.	360	360	360	360	360	360
R-squared	0.9625	0.2370	0.9764	0.9625	0.5764	0.9747

### 5.2 Foreign direct investment

FDI is one of the main driving forces for economic growth [55]. Neoclassical growth theory believes that FDI can only affect economic growth in the short term, and long-term growth mainly depends on exogenous factors. The new economic growth theory first proves that foreign direct investment can affect the long-term economic growth [56]. Foreign direct investment can affect the economy of the importing country in various ways, such as increasing the capital accumulation of the host country and influencing the economic growth of the host country through the competition effect, factor allocation effect, and foreign trade effect [57, 58]. To verify the impact of IIT on the structural power of added value through foreign direct investment, this paper makes a regression between IIT and foreign direct investment, and the results are shown in column (2) of Table 5.

The regression results show that IIT has a significantly positive effect on foreign direct investment, indicating that IIT promotes foreign direct investment, which in turn promotes the rise of a country’s structural power of added value. The coefficients corresponding to β_2_, γ_2_, and η_3_ in the model of the inter-mediation effect of FDI are significant in Table 5 (4)–(6), so the mediating effect exists. The magnitude of this effect is 0.122, accounting for about 7.0% of the total effect of IIT on the structural power of value added. This result suggests that there is a mechanism that exists for IIT to promote the climbing of a country’s structural power of value added by boosting the amount of foreign direct investment, and this mechanism can explain 7.0% of the total impact of Tran on the structural power of value added.

## 6. Conclusions and implications

RCEP region is an important undertaker of the fourth IIT and an indispensable part of the global value chain. Based on the TIVA database (OECD-TIVA, 2021), this paper measures and analyzes IIT and the structural power of value-added. These findings were drawn:

(1) This paper innovatively constructs the IIT index which can reflect both the size and direction and calculates the IIT scale of RCEP countries. Also, it constructs the measurement formula of the structural power of value-added to measure the value-added structural power of RCEP countries.
(2) IIT has significantly promoted the structural power of the value-added of RCEP countries. The benchmark results are still valid after adding a series of control variables and a variety of robustness tests.
(3) The influence of IIT on the structural power of value-added has obvious heterogeneity among countries and trade flow directions. Specifically, IIT significantly promotes the structural power of value-added in developed and developing countries. However, the promotion effect in developed countries is greater than in developing countries. IIT has a significant positive role in promoting both the structural power of value-added inward and outward but the impact of IIT on the structural power of value-added inward is greater than the structural power of value-added outward.
(4) The analysis of the influencing mechanism finds that IIT affects a country’s structural power of value-added through the trade openness mechanism and foreign direct investment mechanism.

Based on the above findings, this paper offers the following suggestions for RCEP countries.

(1) RCEP countries should deeply be involved in the global value chain division and actively undertake IIT. RCEP countries should give full play to their role as “transit hubs” connecting developed economies in the global value chain; and based on their comparative advantages actively adjust their industrial structure, strengthen their international competitiveness of products, and enhance their structural power of value-added.
(2) RCEP countries should enhance human capital and capital intensity, increase R&D investment, and improve innovation capability. As all factors are important, so are the factors affecting the structural power of value-added, just as shown in the empirical baseline results in chapter 4. In detail, RCEP countries should focus on the accumulation of human capital, improve the level of national education, convert labor cost advantage into a human capital advantage, and improve labor production efficiency. Furthermore, most RCEP countries are developing countries, which means that capital intensity is an important factor restricting economic development. So, RCEP countries should pay attention to capital accumulation. Finally, science and technology are the primary productive forces, however developing countries in RCEP generally have low R&D investment, which restricts the speed and quality of economic growth. All in all, RCEP countries should increase R&D investment, encourage innovation, transform into innovative countries, and enhance their national growth potential and vitality.
(3) RCEP should be taken as an opportunity to build a high-level open platform. After the establishment of RCEP, tariff, and non-tariff barriers will be greatly reduced, and the regional flow of factors will be effectively promoted, which is conducive to expanding the growth space of intra-regional and external trade and enhancing the influence and radiation of RCEP regional trade.

## Supporting information

S1 File (XLSX)

## References

[1] Li F., Zhang K., Yang P., Jiao J., Yin Y., Zhang Y., et al. (2022). Information exposure incentivizes consumers to pay a premium for emerging pro-environmental food: Evidence from China. Journal of Cleaner Production, 363, 132412.

[2] Hilpert, H. G. (2022). The Regional Comprehensive Economic Partnership Agreement and Europe: Impact and Implications. Economic Research Institute for ASEAN and East Asia.

[3] Goswami G. G., Khan F., Labiba K., Achol F., Saha T. K., & Zulfikar A. (2022). Should Bangladesh join Regional Comprehensive Economic Partnership (RCEP)? The gravity explanation of Bangladesh dilemma. International Journal of Emerging Markets, (ahead-of-print).

[4] Ito Tadashi, Rotunno Lorenzo, and Vézina Pierre–Louis. 2017. “Heckscher–Ohlin: evidence from virtual trade in value added.” Review of International Economics, vol. 25, no. 3, pp. 427–446.

[5] Fernandes Ana Margarida, Kee Hiau Looi, and Winkler Deborah. 2020. “Determinants of Global Value Chain Participation: Cross-Country Evidence.” The World Bank Economic Review, vol. 36, no. 2, pp. 329–360.

[6] Caporaso J. A. (1978). Introduction to the special issue of International Organization on dependence and dependency in the global system. International Organization, 32(1), 1–12.

[7] Guzzini S. (1993). Structural power: the limits of neorealist power analysis. International Organization, 47(3), 443–478.

[8] Strange, S. (1994). Who governs? Networks of power in world society.5-17

[9] Gill S. R., & Law D. (1989). Global hegemony and the structural power of capital. International studies quarterly, 33(4), 475–499.

[10] Wellman B. (1997). Structural analysis: From method and metaphor to theory and substance. Contemporary Studies in Sociology, 15, 19–61.

[11] Grewal D. S. (2008). Network power: The social dynamics of globalization. Yale University Press.

[12] Oatley T., Winecoff W. K., Pennock A., & Danzman S. B. (2013). The political economy of global finance: A network model. Perspectives on Politics, 11(1), 133–153.

[13] Gereffi G., Humphrey J., Kaplinsky R., & Sturgeon T. J. (2001). Introduction: globalisation, value chains and development. IDS Bulletin, 32(3), 1–8.

[14] Backer, K. D., & Sébastien Miroudot. (2013). Mapping global value chains. OECD Trade Policy Papers.

[15] Amador J., & Cabral S. (2016). Global value chains: A survey of drivers and measures. Journal of Economic Surveys, 30(2), 278–301.

[16] Mansfield E. D., & Pollins B. M. (Eds.). (2009). Economic interdependence and international conflict: New perspectives on an enduring debate. University of Michigan Press.

[17] Savona Maria, and Schiattarella Roberto. 2004. “International Relocation of Production and the Growth of Services: The case of the "Made in Italy" Industries.” Transnational Corporations, vol. 13, no. 2, pp. 57–76.

[18] Li Yanmei, Sun L., Zhang H., Liu T., & Kai F. (2018). Does industrial transfer within urban agglomerations promote dual control of total energy consumption and energy intensity? Journal of Cleaner Production.

[19] Xie H., Chen Q., Lu F., Wu Q., & Wang W. (2018). Spatial-temporal disparities, saving potential and influential factors of industrial land use efficiency: A case study in urban agglomeration in the middle reaches of the Yangtze River. Land use policy, 75, 518–529.

[20] Liu W. (2015). Research on Gradient Transfer of China’s Processing Trades Industries. Journal of Financial Risk Management, 4(03), 216.

[21] Sun Xiaohua, Xun Guo, and Wang Xun. 2018. “Industrial Relocation, Elements Agglomeration and Regional Economic Development.” Journal of Management World, vol. 34, no. 05, pp. 47–62. (in Chinese).

[22] Zhang S., & Chen C. (2020). Does outward foreign direct investment facilitate China’s export upgrading? China & World Economy, 28(5), 64–89.

[23] Li R., Liu Y., & Bustinza O. F. (2019). FDI, service intensity, and international marketing agility: The case of export quality of Chinese enterprises. International Marketing Review, 36(2), 213–238.

[24] Jiang M., Luo S., & Zhou G. (2020). Financial development, OFDI spillovers and upgrading of industrial structure. Technological Forecasting and Social Change, 155, 119974.

[25] Hao Y., Guo Y., Guo Y., Wu H., & Ren S. (2020). Does outward foreign direct investment (OFDI) affect the home country’s environmental quality? The case of China. Structural Change and Economic Dynamics, 52, 109–119.

[26] Tang L., Zhang Y., Gao J., & Wang F. (2020). Technological upgrading in Chinese cities: The role of FDI and industrial structure. Emerging Markets Finance and Trade, 56(7), 1547–1563.

[27] Razzaq A., An H., & Delpachitra S. (2021). Does technology gap increase FDI spillovers on productivity growth? Evidence from Chinese outward FDI in Belt and Road host countries. Technological Forecasting and Social Change, 172, 121050.

[28] Li M., Pan X., & Yuan S. (2022). Do the national industrial relocation demonstration zones have higher regional energy efficiency? Applied Energy, 306, 117914.

[29] Wang H., & Li J. (2021). Dual effects of environmental regulation on PM2. 5 pollution: Evidence from 280 cities in China. Environmental Science and Pollution Research, 28(34), 47213–47226. doi: 10.1007/s11356-021-14011-4 33893580

[30] Yan S., Peng J., & Wu Q. (2020). Exploring the non-linear effects of city size on urban industrial land use efficiency: A spatial econometric analysis of cities in eastern China. Land Use Policy, 99, 104944.

[31] Song Y., Yang T., Li Z., Zhang X., & Zhang M. (2020). Research on the direct and indirect effects of environmental regulation on environmental pollution: Empirical evidence from 253 prefecture-level cities in China. Journal of Cleaner Production, 269, 122425.

[32] Chen, C., Zhao, H., & Zhou, Y. (2017). Foreign direct investment and wage inequality: Evidence from the People’s Republic of China (No. 734). ADBI Working Paper.

[33] Zhang, Shaojun, and Liu, Zhibiao. 2009. “Industry Transference of GVC model——Force, Influence and Inspiration for China’s Industrial Upgrading and Balanced Development of Areas.” China Industrial Economics, no. 11, pp. 5–15.

[34] Liu, Hongguang, Liu, Weidong, and Liu, Zhigao. 2011. “The Quantitative Study on Inter-regional Industry Transfer.” China Industrial Economics, no. 6, pp. 79–88.

[35] Wang Shuli, and Wu Yongliang. 2017. “International Industry Transfer of GVC Model——Based on Empirical Analysis of the Added Value of Trade.” Journal of International Trade, no. 5, pp. 14–24.

[36] Zhao X., & Yin H. (2011). Industrial relocation and energy consumption: Evidence from China. Energy Policy, 39(5), 2944–2956.

[37] Johnson Robert C., and Noguera Guillermo. 2012. “Accounting for Intermediates: Production Sharing and Trade in Value Added.” Journal of International Economics, vol. 86, no. 2, pp. 224–236.

[38] Koopman R., Wang Z., & Wei S. J. (2012). Estimating domestic content in exports when processing trade is pervasive. Journal of Development Economics, 99(1), 178–189.

[39] Antràs Pol, and Chor Davin. 2022. Handbook of International Economics, Volume 5—Chapter 5, Global Value Chains, pp.297–376.

[40] Pang Xun and He Qingqian. 2021. “Structural Power and the Evolution of the International System.” Social Sciences in China, no. 9, pp. 26–46+204–205. (in Chinese).

[41] Karaalp-Orhan, H. S. (2016, October). Human capital, labor productivity and economic growth: The bounds and causality tests for Turkey. In ICPESS (international congress on politic, economic and social studies) (No. 1).

[42] AlKathiri N. (2022). Labour productivity growth and convergence in manufacturing: A nonparametric production frontier approach. Applied Economics, 54(4), 406–429.

[43] Qin B., Liu L., Yang L., & Ge L. (2022). Environmental regulation and employment in resource-based cities in China: The threshold effect of industrial structure transformation. Frontiers in Environmental Science, 10, 47.

[44] Liu J., Hu Y., Xie J., & Li B. (2022). Does cultural diversity contribute to the sustainable development of trade? Empirical evidence from 288 Chinese cities. Growth and Change, 53(1), 432–451.

[45] Yang X., Zhang H., & Li Y. (2022). High-speed railway, factor flow and enterprise innovation efficiency: An empirical analysis on micro data. Socio-Economic Planning Sciences, 82, 101305.

[46] Xie Z., Wu R., & Wang S. (2021). How technological progress affects the carbon emission efficiency? Evidence from national panel quantile regression. Journal of Cleaner Production, 307, 127133.

[47] Shahzad U., Madaleno M., Dagar V., Ghosh S., & Doğan B. (2022). Exploring the role of export product quality and economic complexity for economic progress of developed economies: Does institutional quality matter? Structural Change and Economic Dynamics, 62, 40–51.

[48] Qiu Bin, Tang Baoqing, Sun Shaoqin, and Liu Xiuyan. 2014. “Factor Endowment, Institutional Dividend and New Comparative Advantage of Exports.” Economic Research Journal, vol. 49, no. 8, pp. 107–119.

[49] Cui Xinghua. 2021. “Foreign Intermediate Service Investment and Manufacturing Global Value Chain Status: Empirical Analysis based on WIOD Input-output Data.” Business and Management Journal, vol. 43, no, 3, pp. 26–42. (in Chinese).

[50] Baron R. M., & Kenny D. A. (1986). The moderator-mediator variable distinction in social psychological research: conceptual, strategic, and statistical considerations. Chapman and Hall, 51(6), 1173–1182. doi: 10.1037//0022-3514.51.6.1173 3806354

[51] Wang Q., & Wang L. (2021). How does trade openness impact carbon intensity? Journal of Cleaner Production, 295, 126370.

[52] Zaman M., Pinglu C., Hussain S. I., Ullah A., & Qian N. (2021). Does regional integration matter for sustainable economic growth? Fostering the role of FDI, trade openness, IT exports, and capital formation in BRI countries. Heliyon, 7(12), e08559. doi: 10.1016/j.heliyon.2021.e08559 34917822 PMC8669309

[53] Kong Q., Peng D., Ni Y., Jiang X., & Wang Z. (2021). Trade openness and economic growth quality of China: Empirical analysis using ARDL model. Finance Research Letters, 38, 101488.

[54] Alam M. M., & Murad M. W. (2020). The impacts of economic growth, trade openness and technological progress on renewable energy use in organization for economic co-operation and development countries. Renewable Energy, 145, 382–390.

[55] Chen S., Zhang H., & Wang S. (2022). Trade openness, economic growth, and energy intensity in China. Technological Forecasting and Social Change, 179, 121608.

[56] Hamid I., Alam M. S., Murshed M., Jena P. K., Sha N., & Alam M. N. (2021). The roles of foreign direct investments, economic growth, and capital investments in decarbonizing the economy of Oman. Environmental Science and Pollution Research, 1–17. doi: 10.1007/s11356-021-17246-3 34782975

[57] Dinh T. T. H., Vo D. H., The Vo A., & Nguyen T. C. (2019). Foreign direct investment and economic growth in the short run and long run: Empirical evidence from developing countries. Journal of Risk and Financial Management, 12(4), 176.

[58] Esquivias M. A., & Harianto S. K. (2020). Does competition and foreign investment spur industrial efficiency: firm-level evidence from Indonesia. Heliyon, 6(8), e04494. doi: 10.1016/j.heliyon.2020.e04494 32904310 PMC7452401

